# Case Report: Physiological Stress Responses to Repeated, Standardized Short-Distance Transport in a Transport-Experienced Horse

**DOI:** 10.3390/ani16091293

**Published:** 2026-04-22

**Authors:** Lore Pellens, Louis Freson, Johan Buyse, Bert Driessen

**Affiliations:** 1 Animal Welfare Solutions, 3538 Paal, Belgium; lore@animalwelfaresolutions.com (L.P.); lodewijkfreson@skynet.be (L.F.); 2 Agricultural Buildings Research, Department of Biosystems, KU Leuven, 3001 Heverlee, Belgium; 3 Laboratory of Humane Biology, Department of Physiology, KU Leuven, 3000 Leuven, Belgium; 4 Laboratory of Livestock Physiology, Department of Biosystems, KU Leuven, 3001 Heverlee, Belgium; johan.buyse@kuleuven.be; 5 Department of Morphology, Medical Imaging, Orthopaedics, Physiotherapy and Nutrition, Laboratory of Morphology, Faculty of Veterinary Medicine, Ghent University, 9820 Merelbeke, Belgium

**Keywords:** horse, transport, training, heart rate, cortisol, case report

## Abstract

Transport is a fundamental component of equestrian training and competition. However, even horses with extensive transport experience may exhibit physiological stress responses during routine travel. This case report describes physiological stress responses in a transport-experienced 10-year-old Belgian Warmblood gelding subjected to repeated, standardized short-distance transport. The horse was transported on 17 occasions along a fixed route to a riding school for dressage training using the same vehicle, handler, and protocol to minimize environmental variability. Physiological stress markers were assessed using continuous heart rate monitoring and salivary cortisol sampling at standardized time points before, during, and after each transport and training session. Salivary cortisol concentrations increased during transport and remained slightly elevated relative to preloading reference values throughout the transport–training–return sequence. Heart rate was elevated relative to typical resting values prior to loading, peaked during training, and remained moderately elevated during subsequent transport phases. Longitudinal visualization across repeated transport events revealed a gradual decline in cortisol concentrations after approximately ten transport events, suggestive of partial physiological habituation. However, the occurrence of a physiological outlier during the fifth transport event underscores that even in experienced horses, acute stress responses can occur independently of the general habituation trend. These findings indicate that repeated short-distance transport can elicit measurable physiological stress responses in transport-experienced horses and that habituation may be incomplete despite standardized conditions and familiarity with the procedure. Taken together, these results challenge the assumption that routine transport is minimally stressful, highlighting the importance of individualized transport management, adequate recovery periods, and ongoing welfare monitoring.

## Simple Summary

Horses used in sport and competition are frequently transported as part of their regular routine. While it is often assumed that experienced horses adapt to transport over time and cope with it without difficulty, this case report shows that measurable physiological stress responses can persist even in a well-experienced horse. A 10-year-old Belgian Warmblood gelding was transported on 17 occasions along a fixed 13 km route to a riding school for dressage training. Each transport event followed an identical protocol, with the same vehicle, driver, handler, and departure time, to keep conditions as consistent as possible. Stress was assessed using two complementary indicators: continuous heart rate monitoring and repeated salivary cortisol sampling at seven defined stages before, during, and after each transport and training session. Over the course of the study, cortisol concentrations gradually declined after approximately the tenth transport event, suggesting partial habituation to the repeated procedure. However, one transport event produced a pronounced spike in cortisol that was nearly five standard deviations above the study mean, demonstrating that even experienced horses can experience acute stress responses on individual occasions without any identifiable cause. Heart rate did not exhibit a comparable longitudinal pattern as cortisol, highlighting the importance of monitoring both indicators rather than relying on a single measure. These findings suggest that routine short-distance transport should not be considered stress-free simply because a horse is experienced with travel. Individual monitoring, consistent handling, adequate recovery time, and attention to loading procedures remain important components of responsible equine transport management.

## 1. Introduction

Horses used in training and competition are routinely transported, exposing them to a combination of physical and psychological challenges, including confinement, vehicle motion, environmental novelty, and social separation. Transport-related stress has been associated with changes in physiological parameters, behavioral responses, and welfare outcomes in horses, making it a persistent topic of interest in equine science and welfare research [1,2].

Physiological indicators such as heart rate and salivary cortisol concentration are commonly used to assess stress responses in horses. They reflect complementary aspects of the stress response but differ in their temporal dynamics and physiological specificity. Heart rate responds rapidly to both physical exertion and psychological stimuli, including anticipatory arousal, whereas cortisol reflects activation of the hypothalamic–pituitary–adrenal axis (HPA) with a delayed response profile in response to psychological and physiological challenges [1,3,4,5]. Studies using novel object tests and other psychological challenge paradigms further demonstrate that heart rate can increase independently of exercise-related load [1]. In contrast, cortisol responses may reflect both exercise-induced physiological activation and psychological stress, requiring contextual interpretation [2,6]. Transport-naive horses typically show pronounced physiological stress responses that may attenuate with repeated exposure, suggesting habituation [7]. However, even horses with transport experience can exhibit measurable stress responses, particularly during loading procedures and transport itself [8,9,10].

Salivary cortisol measurement offers a practical and minimally invasive approach for assessing stress in horses, making it well suited for repeated sampling during transport-related procedures [11]. Salivary cortisol reflects the free, biologically active fraction of circulating cortisol and responds rapidly to acute stressors [12,13]. A strong correlation between salivary and serum cortisol concentrations has been demonstrated in horses, supporting its validity as an indicator of HPA axis activation [12].

Most studies investigating transport-related stress in horses focus on single transport events or compare transport-naive and experienced animals. Longitudinal data documenting within-individual physiological responses across repeated, short-distance transport events under standardized conditions remain particularly scarce. The majority of published work examines longer transport durations where physical fatigue and dehydration are primary stressors [9,14]. This single-subject case report addresses this gap by describing physiological stress responses in a transport-experienced horse subjected to 17 repeated short-distance transport events along a fixed route. By combining salivary cortisol analysis and continuous heart rate monitoring across defined stages of transport and training, this study provides a detailed within-subject characterization of physiological responses and temporal patterns across repeated exposures under real-world conditions. Rather than attempting to isolate transport stress from training-related physiological activation, this study intentionally characterizes the cumulative stress response across the complete transport–training–return sequence. This approach reflects the applied reality of equestrian sport, in which transport and training represent an integrated physiological challenge rather than independent stressors.

The aim was not to study initial acclimatization but to characterize physiological responses under routine, real-world transport conditions in an already transport-experienced horse. This study provides a methodological basis for future controlled studies on habituation processes.

## 2. Materials and Methods

### 2.1. Study Animal

A 10-year-old Belgian Warmblood gelding accustomed to routine dressage training and regular road transport was included in this case report. The horse was housed individually in a straw-bedded loose box at a private stable in Belgium (location A) and maintained on a consistent management regimen throughout the study period. The diet consisted of concentrates and hay provided twice daily, with ad libitum access to water. Prior to enrollment, the horse was considered clinically healthy based on routine veterinary care and the absence of known medical conditions or prior transport-related complications. No changes to housing, feeding, training intensity, or transport routine were introduced for the purpose of this study. Data were collected in the winter (between December and February).

### 2.2. Transport Protocol

The horse was transported by road on 17 occasions at a frequency of approximately twice weekly. Each transport event consisted of an outward journey from the stable (location A) to a riding school (location B; distance: 13 km; duration: approximately 20 min), followed by a dressage training session and a return journey to location A.

All transports were conducted using the same trailer (Atec Technoline; internal dimensions: 3.12 m (length) × 1.28 m (width) × 2.22 m (height)) towed by the same vehicle (BMW 318d) and driven by the same driver with practical experience in routine horse transport. Loading was performed via a rear ramp (1.28 m × 1.43 m) positioned 10 m from the stable. The loading ramp was positioned at an approximate angle of 13°, which is within recommended limits for equine loading [5]. To minimize procedural variability, all handling and loading procedures were performed by the same experienced handler, and transport was consistently initiated at approximately 10:00. Environmental conditions within the trailer (temperature, humidity, noise, vibration) were not instrumented during this study. The horse’s head was tied in a manner that ensured the preservation of its mobility, allowing it to move its head freely in the left and right directions, as well as below its withers.

Loading time was defined as the interval from leading the horse out of the stable until it reached its final position inside the trailer, facing the direction of travel. No feed or water was provided during transport, and no intermediate stops were made. Upon arrival at location B, the horse was immediately unloaded and trained in dressage for an average duration of 49 ± 7 min. The reported training duration refers to the active riding period and excludes time required for saddling, bridling, and untacking. After training, the horse was reloaded and transported back to location A, where it was immediately unloaded and returned to its stable. No period of free movement or turnout was provided prior to the recovery sampling. Durations of all transport and handling stages were recorded using a stopwatch.

### 2.3. Physiological Sampling Protocol

Physiological measurements were obtained at seven standardized time points corresponding to predefined stages of the transport and training sequence: (1) pre-loading reference, 30 min before loading at location A; (2) immediately after loading at location A; (3) after outward transport, upon arrival at location B; (4) after the dressage training session; (5) after loading at location B for the return journey; (6) after return transport, upon arrival at location A; and (7) recovery assessment, 30 min after final unloading at location A. Both salivary cortisol and heart rate data were referenced to these same seven stages across all 17 transport events. Since heart rate was continuously recorded, the average heart rate in each stage was used in the analysis.

### 2.4. Salivary Cortisol

Salivary cortisol reflects free cortisol concentrations in blood due to rapid diffusion into the salivary glands, with changes typically detectable within minutes following HPA axis activation [11,12]. Saliva samples were collected at the seven time points described in Section 2.3. Samples were obtained using cotton rolls (Salivette, Sarstedt, Germany) placed in the left corner of the horse’s mouth and rotated for one minute [15]. Samples were stored at 4 °C and centrifuged within four hours of collection (1000 *g* for 10 min), after which saliva was extracted and stored at −20 °C until analysis.

Cortisol concentrations were quantified using a validated direct enzyme immunoassay (DetectX^®^ Cortisol Enzyme Immunoassay Kit, Arbor Assays^®^, Ann Arbor, MI, USA) with a detection limit of 45.4 pg/mL and intra- and inter-assay coefficients of variation of 6.0% and 7.2%, respectively [16]. The immunoassay was previously validated for equine saliva [17,18].

### 2.5. Heart Rate

Heart rate was continuously recorded using a Polar Equine RS800CX Science system, Polar Electro Oy, Kempele, Finland [19]. The recording unit was secured to the horse’s thorax using an elastic girth approximately 30 min before loading, corresponding to the first standardized stage. Heart rate data were recorded at five-second intervals from the pre-loading period until 30 min after final unloading at location A. Data were processed using Polar ProTrainer 5 Equine Edition software, the mean heart rate per stage was calculated as the average of all 5-s interval recordings within that stage. The minimum and peak heart rate values were also calculated for each of the same seven standardized stages used for salivary cortisol sampling.

### 2.6. Ethical Approval and Consent

All procedures were observational in nature and coincided with the horse’s routine transport and training activities. The study was conducted in accordance with Council Directive 86/609/EEC (now replaced by Directive 2010/63/EU) for the protection of animals used for scientific and educational purposes [20,21]. Informed consent was obtained from the owner prior to data collection.

### 2.7. Statistical Analysis

Data analysis was performed using R (version 4.5.1) [22]. Given the single-subject design, no inferential statistical tests were applied. Analyses were used solely to describe and visualize within-individual temporal variation across repeated transport events, rather than to infer population-level effects.

Continuous heart rate and salivary cortisol data were analyzed for longitudinal trends using LOESS (locally estimated scatterplot smoothing) with the span parameter equal to 0.5. These smoothed curves were used for descriptive purposes only. Individual transport events were screened for values deviating substantially from the within-study distribution. As a descriptive criterion, values exceeding two standard deviations from the study mean were flagged as physiological outliers. This threshold is applied as a practical heuristic within a single-subject context and does not constitute a validated inferential criterion.

## 3. Results

### 3.1. Timeline and Stage Durations

Table 1 summarizes the 17 transport events conducted over a 10-week study period. Each event followed an identical seven-stage protocol as defined in Section 2.3. Transport duration remained highly consistent (outward transport: 22 ± 2 min; return transport: 22 ± 1 min). Loading time differed substantially between locations: loading at location A before training was considerably longer (70 ± 58 s) than loading at location B after training (22 ± 10 s). Pre-training loading time showed substantial variability (coefficient of variation ≈ 83%).

### 3.2. Salivary Cortisol

**Figure 1:**
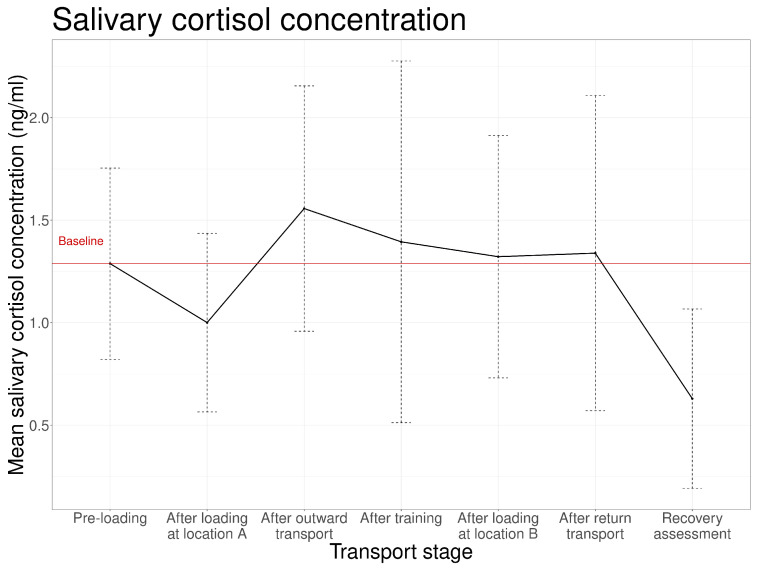
Mean salivary cortisol concentrations (±the standard deviation) for each stage (*n* = 17 transport events).

The mean salivary cortisol concentration after the pre-loading reference period was 1.33 ± 0.49 ng/mL (Figure 1). Cortisol concentrations increased after outward transport and subsequently decreased after training. After final unloading, the level of salivary cortisol was lower compared to that in the pre-loading reference period. The mean cortisol level measured after outward transport was slightly higher than those observed after return transport. The mean salivary cortisol concentration following loading at location A and location B exhibited a larger difference, the concentration diminished after loading at location A, reaching levels below pre-loading values. Conversely, the concentration remained slightly above pre-loading values after loading at location B.

**Figure 2:**
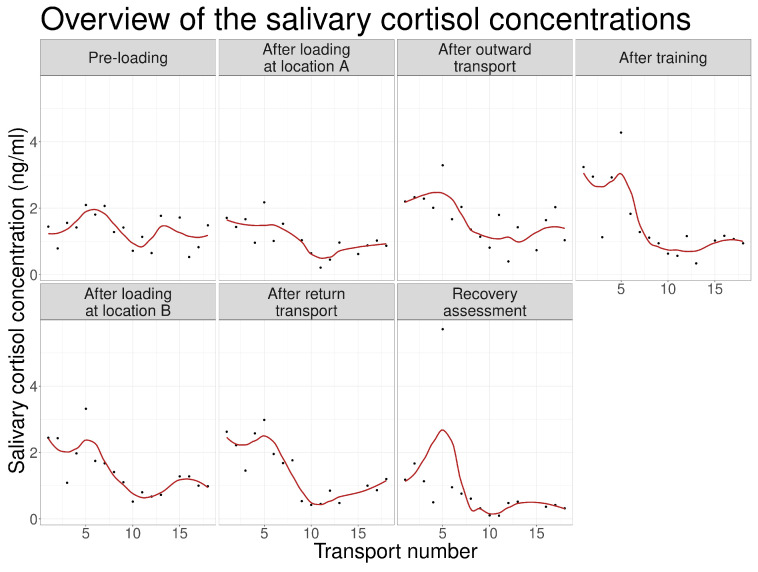
Salivary cortisol concentrations across all 17 transport occasions with LOESS trend lines.

When visualized across the 17 transport occasions, salivary cortisol concentrations exhibited a gradual downward trend over successive events (Figure 2). LOESS smoothing indicated that this decline became most apparent after approximately the tenth transport event, after which concentrations stabilized at lower values than those observed during earlier events.

A notable physiological outlier occurred during the fifth transport event, where salivary cortisol reached a study-wide maximum of 5.71 ng/mL. Given the overall study mean of 1.36 ng/mL and standard deviation of 0.88 ng/mL, this value deviates from the mean by nearly five standard deviations, representing a pronounced acute stress response well outside the general habituation trend.

### 3.3. Heart Rate

**Figure 3:**
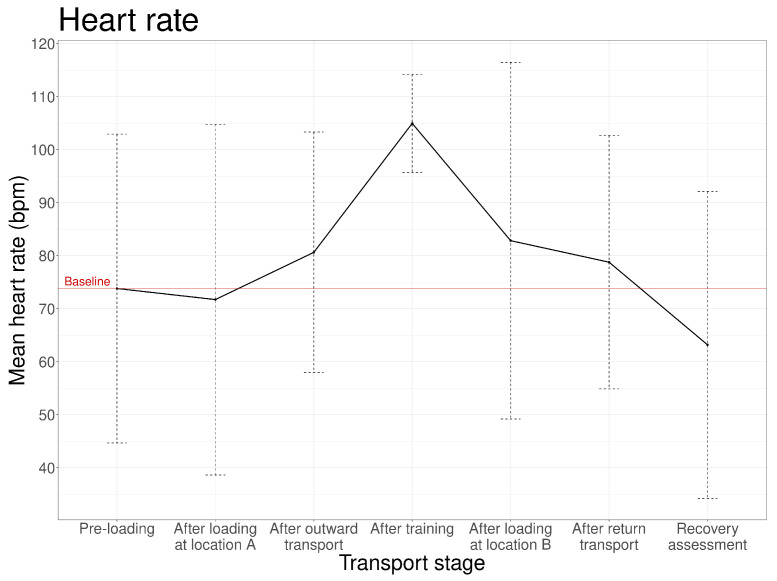
Mean heart rate (±the standard deviation) for each transport stage (n = 17 transport events).

Mean heart rate after the pre-loading reference period was 73.80 ± 29.10 bpm (Figure 3), substantially exceeding typical resting values for horses (28–40 bpm [23]). Heart rate increased substantially after training (105 ± 9.23 bpm), reflecting the physical demands of dressage work, and subsequently decreased but remained elevated relative to pre-loading reference values after subsequent loading and transport stages.

No systematic differences in mean heart rate were observed between outward and return transport stages, nor between loading at location A and loading at location B. Following training, heart rate decreased but remained elevated relative to pre-loading reference values after subsequent loading and transport.

**Figure 4:**
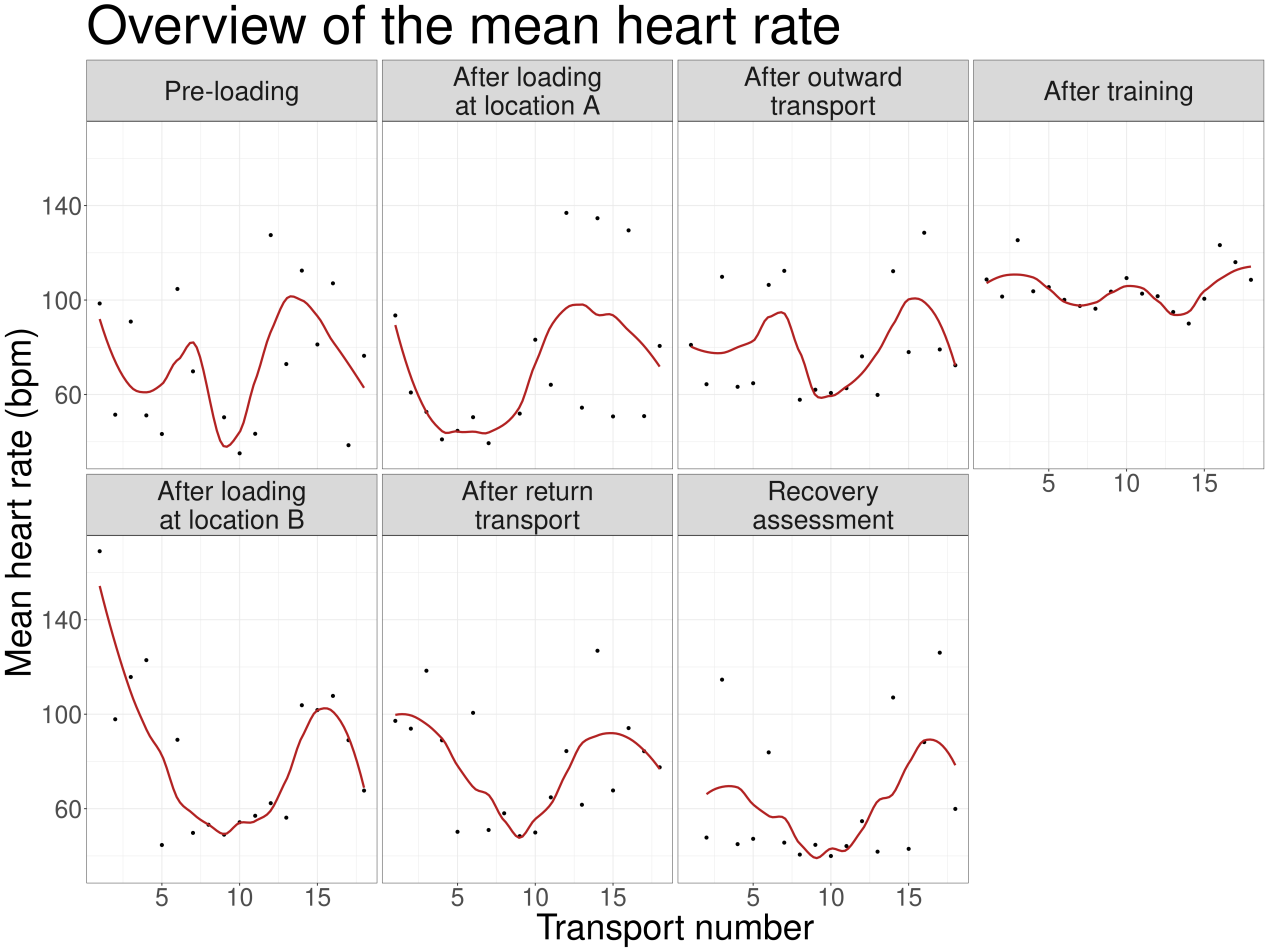
Mean heart rate across all 17 transport events with LOESS trend lines.

Across repeated transport events, mean heart rate values displayed variable temporal patterns (Figure 4). LOESS trend lines illustrated an initial reduction at several stages during the first half of the study, followed by a partial increase in later events.

Given the single-subject design, no formal correlation analysis between heart rate and salivary cortisol was performed. Interpretation is therefore based on descriptive and visual comparison of temporal patterns.

## 4. Discussion

The main finding of this case study is that repeated short-distance transport was associated with persistent physiological stress responses in this transport-experienced horse, with evidence of partial but incomplete habituation over time.

### 4.1. Stage Duration and Loading Behavior

Transport duration was highly consistent across the study, confirming stable travel conditions. Loading time before training was substantially longer and more variable than loading after training, despite identical equipment and handling. This difference may reflect multiple interacting factors. Pre-training loading involves separation from the familiar stable environment and may therefore be associated with longer loading times [24]. Post-training loading signals return to the home environment and may be associated with shorter loading times, potentially reflecting anticipatory responses. Physical exertion during training may also contribute to reduced loading time through fatigue.

Importantly, shorter loading times should not be interpreted as reduced stress, as behavioral expression may be constrained by fatigue rather than reflecting improved welfare. The high day-to-day variability in pre-training loading times suggests that responses may be influenced by occasion-specific factors not captured by physiological indicators alone. Individual horses may adopt different adaptive strategies to repeated exposure, including habituation or sensitization, and behavioral responses alone may not reliably reflect underlying mental state [25,26,27].

As behavioral responses during loading were not systematically recorded, interpretation of loading time differences should be considered indirect and may reflect multiple interacting factors rather than specific behavioral states.

### 4.2. Salivary Cortisol

Pre-loading reference cortisol concentrations were elevated compared to some published baseline values but remained within reported ranges for horses [12,28,29]. Direct comparison across studies is limited by differences in assay methods, sampling protocols, time of day, and management conditions. In this study, all samples were processed using the same validated assay protocol, making systematic analytical variation unlikely. Elevated pre-loading cortisol concentrations may reflect anticipatory arousal associated with a predictable routine, as well as circadian influences due to morning sampling [2,30]. In addition, pre-loading reference cortisol levels may be influenced by broader management-related factors such as housing conditions, turnout, social interaction, and feeding or foraging opportunities, all of which can affect equine welfare and physiological stress responses [2,31,32,33].

Cortisol concentrations increased after transport and remained elevated throughout the transport–training–return sequence, before decreasing after final unloading. This pattern is consistent with previous findings in transport-experienced horses and contrasts with the more prolonged recovery observed in transport-naive horses [3,7,34]. The absence of a detectable increase immediately after loading is likely related to the temporal dynamics of cortisol secretion, as salivary cortisol typically increases 20–30 min after stressor onset [35]. Given the short duration of loading, this delay likely explains the lack of response at that stage. The persistence of elevated cortisol across subsequent stages may reflect a cumulative response to transport-related and exercise-related stimuli, which cannot be disentangled within the present design [6].

### 4.3. Heart Rate

Heart rate during the pre-loading reference period exceeded typical resting values for horses [23]. This likely reflects anticipatory and handling-related arousal rather than true resting heart rate. The attachment of the monitoring equipment prior to loading, as well as routine stable activity, may also have contributed to elevated values.

The substantial variability observed after pre-loading heart rate suggests that day-to-day differences in behavioral state or pre-transport activity may have influenced these measurements. Anticipatory responses associated with predictable events are a plausible explanation, although physical activity related to handling and preparation may also contribute [4,36].

Mean heart rate was highest after training, consistent with the physical demands of exercise, and decreased thereafter but remained elevated relative to pre-loading values after subsequent transport stages. These responses likely reflect a combination of physical exertion, balance adjustments during transport, and psychological arousal.

### 4.4. Temporal Patterns Across Repeated Transport Events

Longitudinal visualization revealed a gradual decline in salivary cortisol concentrations across repeated transport events, particularly after approximately ten events, which may indicate partial physiological habituation. In contrast, heart rate patterns showed greater variability over time, with an initial reduction followed by a partial increase in later events. This divergence may suggest that adaptation of the HPA axis and autonomic responses occurs at different rates [7]. A descriptive comparison of stage-specific responses suggests that the relationship between heart rate and cortisol varied across the transport–training sequence. Periods of apparent alignment were observed following transport and training, where both parameters were elevated. However, divergence was observed at specific stages. During training, heart rate reached its highest values, whereas salivary cortisol did not show a proportional increase, which may indicate a stronger influence of physical exertion on heart rate. Conversely, during post-transport stages, heart rate decreased while cortisol remained elevated, which may suggest a more prolonged response of the HPA axis. These observations should be interpreted cautiously, as they are based on a single subject and descriptive analysis only.

### 4.5. Welfare Implications and Practical Recommendations

In this individual horse, physiological indicators remained elevated across the transport–training–return sequence, suggesting that routine short-distance transport may be associated with measurable physiological activation even in a transport-experienced horse. The occurrence of an acute cortisol outlier during one transport event further illustrates that responses may vary substantially between occasions, even under apparently stable conditions.

These observations highlight the potential value of individual monitoring, as responses to repeated transport may not follow a uniform pattern. Management strategies such as consistent handling, adequate recovery periods, and attention to loading procedures may support welfare by reducing cumulative physiological demands [5].

### 4.6. Study Limitations

Several limitations should be considered when interpreting these observations. As a single-subject case study, the findings are specific to this individual horse and cannot be generalized to other horses or transport conditions. The absence of baseline measurements on non-transport days limits the ability to distinguish transport-related responses from anticipatory or routine-related physiological activation. Environmental conditions within the trailer, including temperature, humidity, noise, and vibration, were not monitored and may have influenced physiological responses. Driving conditions such as traffic density and braking patterns were also not recorded. In addition, behavioral responses during loading were not systematically quantified, limiting interpretation of loading time differences. Finally, heart rate variability was not assessed, which may have provided further insight into autonomic regulation [37].

### 4.7. Implications for Future Research

While the observations described here are specific to a single horse, they may inform the design of future studies investigating transport-related stress. The standardized protocol used in this study, combining repeated salivary cortisol sampling with continuous heart rate monitoring across defined stages, provides a practical framework for assessing physiological responses under real-world conditions. Future research in larger populations, incorporating controlled variation in transport conditions, behavioral observations, and additional physiological measures such as heart rate variability, would allow further evaluation of the relative contributions of transport, training, and environmental factors to equine stress responses.

## 5. Conclusions

This case report demonstrates that repeated short-distance transport elicited persistent physiological stress responses in a transport-experienced gelding despite standardized conditions, consistent handling, and route familiarity. Salivary cortisol increased during transport and remained elevated throughout the procedure, while heart rate exceeded typical resting values even prior to loading and peaked after training. Longitudinal visualization suggested partial habituation across repeated exposures, although physiological markers remained elevated relative to pre-loading references, indicating incomplete adaptation.

The divergence between cortisol and heart rate trajectories across the study period highlights that these indicators capture complementary stress dimensions and should ideally be assessed in combination. The occurrence of an acute stress outlier during an otherwise stable study period further illustrates that individual variability and unpredictable stress responses can arise even in experienced horses under standardized conditions.

These observations challenge assumptions that routine transport becomes minimally stressful through habituation alone, and highlight the importance of individualized transport management, adequate recovery strategies, and ongoing physiological monitoring to support equine welfare.

## Figures and Tables

**Table 1:** Summary timeline of transport events and key physiological observations.

Event	Date	Key Observations
Baseline	Prior to December	Horse was transport-experienced with a regular training routine.
Transport 1–9	December–January	Higher cortisol concentrations across stages (physiological outlier at transport event 5); variable loading times before and after training; elevated heart rate from pre-loading reference.
Transport 10	Mid-January	LOESS trend lines indicate inflection toward declining cortisol concentrations across all sampling stages.
Transport 11–17	Late-January–February	Cortisol stabilized at lower values; heart rate showed variable pattern with some increasing trends.
Physiological recovery	Throughout study	Cortisol returned toward pre-loading reference within 30 min post-transport; heart rate decreased rapidly post-training but remained slightly elevated.

## Data Availability

The raw data supporting the conclusions of this article will be made available by the authors, without undue reservation.
